# DNA methylation-mediated high expression of CCDC50 correlates with poor prognosis and hepatocellular carcinoma progression

**DOI:** 10.18632/aging.204899

**Published:** 2023-08-07

**Authors:** Chunhong Li, Yingdong Jia, Na Li, Qiang Zhou, Rui Liu, Qiang Wang

**Affiliations:** 1 Department of Oncology, Suining Central Hospital, Suining 629000, Sichuan, P.R. China; 2 Gastrointestinal Surgical Unit, Suining Central Hospital, Suining 629000, Sichuan, P.R. China; 3 Department of Radiation Oncology, Suining Central Hospital, Suining 629000, Sichuan, P.R. China

**Keywords:** CCDC50, DNA methylation, prognostic value, hepatocellular carcinoma, immune cell infiltra

## Abstract

Hepatocellular carcinoma (HCC) is one of the most common and lethal cancer types worldwide. Recent studies found Coiled-coil domain-containing protein 50 (CCDC50) could regulate the nuclear factor kappa-B and p53 signalling pathways in cancer. Nevertheless, the underlying biological function and potential mechanisms of CCDC50 driving the progression of HCC remain unclear. In this study, we found that CCDC50 was up-regulated in HCC, and its higher expression was associated with adverse clinical outcomes and poor clinical characteristics. The results of the Cox regression analysis revealed that CCDC50 was an independent factor for the prognosis of HCC. Meanwhile, we also established a nomogram based on CCDC50 to predict the 1-, 3-, or 5-year survival in HCC patients. Furthermore, we found that DNA hypomethylation results in its overexpression in HCC. In addition, functional annotation confirmed that CCDC50 was mainly involved in the neuroactive ligand-receptor interaction and protein digestion and absorption. Importantly, we found that CCDC50 was highly expressed in HCC cell lines. Depletion of CCDC50 significantly inhibits HCC cell proliferation and migration abilities. This is the first study to identify CCDC50 as a new potential prognostic biomarker and characterize the functional roles of CCDC50 in the progression of HCC, and provides a novel potential diagnostic and therapeutic biomarker for HCC in the future.

## INTRODUCTION

Worldwide, hepatocellular carcinoma (HCC) is one of the major diseases threatening the health of humans [1–3]. Although the diagnosis and treatment of it are becoming more perfect, the prognosis of HCC patients has not been significantly improved [4]. Reliable biomarkers are not only helpful for early diagnosis and prognosis of cancer patients, but also can reduce medical costs [5–7]. Thus, elucidating the specific biomarkers in the process of HCC development has a critical research significance for early diagnosis and personalized medicine in patients.

Coiled-coil domain-containing protein 50 (CCDC50) was first identified as mapping to chromosome 3q28 [8]. As a negative regulator of IFN signalling, it is ubiquitously expressed in human tissues [9]. It has been reported that CCDC50 was enhanced by viral infection, and could inhibit the NF-κB-mediated apoptotic pathway, enhance the viral resistance, and regulate the p53 signalling pathways [8, 10, 11]. Further, CCDC50 could regulate Ras signalling pathway and promote mice HCC [12]. However, the potential role of CCDC50 in human HCC progression remains unclear. Our study aimed to examine the relationships between CCDC50 expression and diverse features in HCC. Furthermore, the CCK8 and transwell assays were employed to determine the biological role of CCDC50 in HCC progression.

## MATERIALS AND METHODS

### Analysis of CCDC50 expression in pan-cancer

GEPIA (http://gepia.cancer-pku.cn/) is a web-based tool that quickly outputs customizable data results, and can be interactively used for analysing gene expression data of the TCGA clinical data and RNA-seq [13]. We analysed the CCDC50 expression across TCGA tumors, and the GTEx data and matched TCGA normal were contained as controls. UALCAN database is a powerful web-portal [14], herein, we detached CCDC50 protein expression via UALCAN web-portal.

### The prognostic and clinical information of CCDC50 in HCC

We used the GEPIA [13] and PrognoScan [15] to examine the prognostic OS, and DSS of CCDC50 in HCC. The gene mutation features of CCDC50 in HCC were analysed via the cBioPortal [16]. We chose the three datasets: “MSK, Clin Cancer Res 2018 [17], MERiC/Basel, Nat Commun. 2022 [18], and TCGA. We explored the TIMER2.0 to estimate the immunological roles of CCDC50 in HCC [19].

### Cell culture, siRNA and qRT-PCR

LIHC cell lines and LO2 cells lines were obtained from ATCC and cultured in RPMI-1640 medium supplemented with 10% FBS. CCDC50 siRNA kits (si-CCDC50#1: 5’- CCGUGCUUAUGCAGAUAGUTT-3’; si-CCDC50#2: 5’- GCAGCAAAUUCCAAGUCAATT-3’) and negative control siRNAs (si-NC: 5’-UUCUCCGAACGUGUC ACG UTT-3’) were purchased from GenePharma. Transfections were performed as previously described [20]. The qRT-PCR assay was conducted as previously documented [20]. The following primer sequences were used in this study: CCDC50: 5’-GACGACGCATTCAGGAGAAGA-3’, 5’-ACTATCTGCATAAGCACGGGTT-3’; β-actin: 5’-GTCTTCCCCTCCATCGTG-3’’, 5’-AGGGTGAGGATGCCTCTCTT-3’.

### Western blot

Western blot assays were fulfilled as described previously [20]. The following primary antibodies were used in this finding: CCDC50 (A17836; Abclonal) and β-actin (sc-47778; Santa Cruz). All the assays were independently repeated thrice.

### Cell proliferation assay

CCK8 assays were fulfilled as described previously [20]. For CCK8 assay, Hep3B and Huh7 cells were cultured in a 96-well plate supplemented with 200 μL of RPMI-1640 medium.

### Correlation between CCDC50 and cancer drug sensitivity

GSCA [21] is an integrated platform combining clinical and small molecular drugs information, it can help us easily analyse CCDC50 expression with drug sensitivity GDSC and CTRP [22, 23].

### Data availability statement

The data from this article can be obtained from the public database The Cancer Genome Atlas (https://portal.gdc.cancer.gov/).

## RESULTS

### CCDC50 was differentially expressed in multiple cancer including HCC

**Figure 1:**
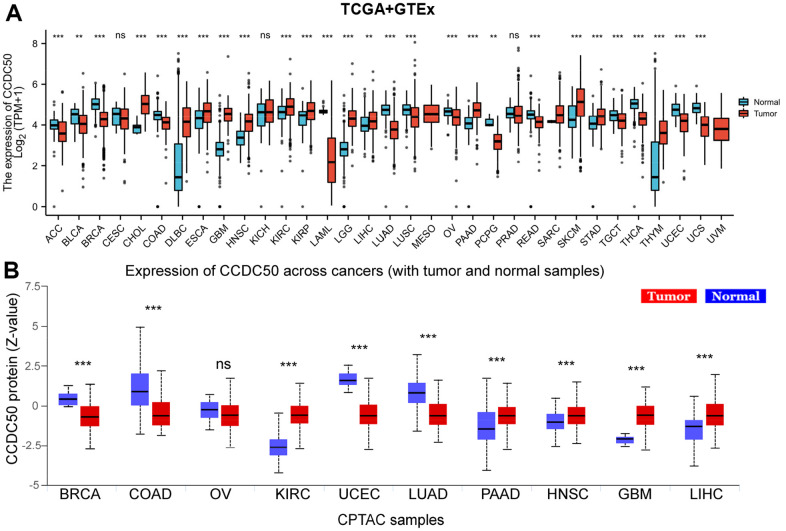
**CCDC50 mRNA and protein expressions between tumour and normal tissues.** (**A**) The CCDC50 expression in pan-cancer analysis via the TCGA/GTEx databases. (**B**) The protein of CCDC50 in pan-cancer analysis from CPTAC samples via the UALCAN web-portal. Z-values represent standard deviations from the median across samples for the given cancer type. ns, p > 0.05; *p < 0.05; **p < 0.01; ***p < 0.001.

Firstly, we combined the TCGA and GTEx databases, and confirmed that the expression of CCDC50 was significantly lower in 14 cancer tissues than normal tissues. In addition, CCDC50 was up-regulated in 12 types of cancer including HCC (Figure 1A). As the main undertaker of life activities, the change of protein expression level is directly related to cancer progression [24]. To determine the protein levels of CCDC50 in different types of cancer, we analysed the UALCAN database and found that the protein expression of CCDC50 was low in 4 types of cancer, whereas it was high in 5 types of cancer including HCC (Figure 1B).

**Figure 2:**
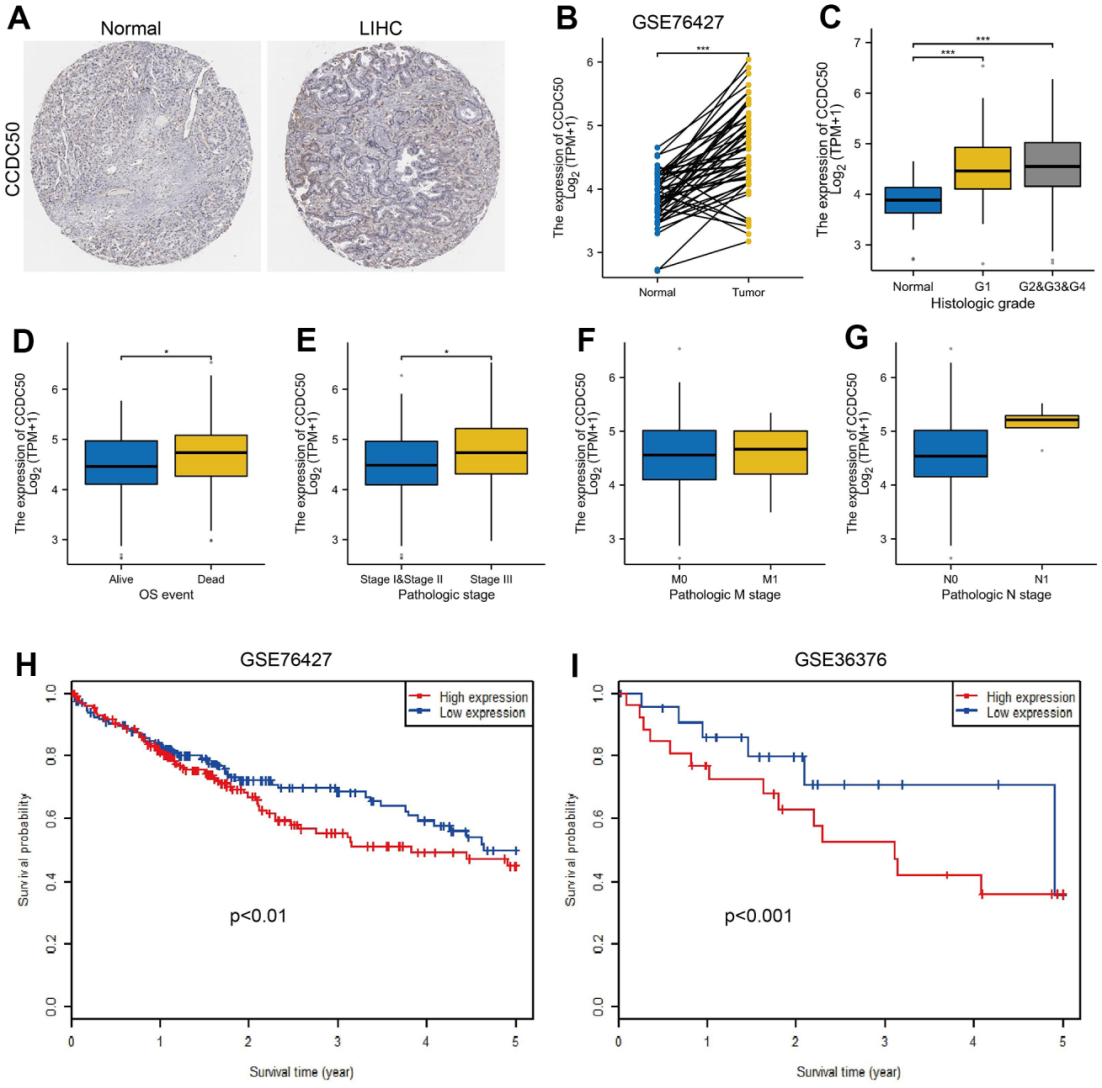
**CCDC50 was highly expressed in HCC.** (**A**) CCDC50 protein was highly expressed in HCC tissues than normal liver tissues examined by HPA database. (**B**) Validation of the expression of CCDC50 in HCC by GEO dataset. (**C**–**G**) Relationships between the expression of CCDC50 and diverse clinical features in HCC. (**H**, **I**) Validation of the prognosis of CCDC50 in HCC by GEO dataset.

To explore CCDC50 expression levels in HCC, we used HPA and GEO datasets to validate its expression in HCC. We showed that CCDC50 was upregulated in HCC (Figure 2A, 2B). Furthermore, we showed that CCDC50 was correlated with adverse clinical features, such as grade, pathological stage and OS event, not related to NM stage (Figure 2C–2G). GEO datasets also confirmed that high CCDC50 expression had worse OS in HCC cancer patients (Figure 2H, 2I).

### Prognosis value of CCDC50 in HCC

**Figure 3:**
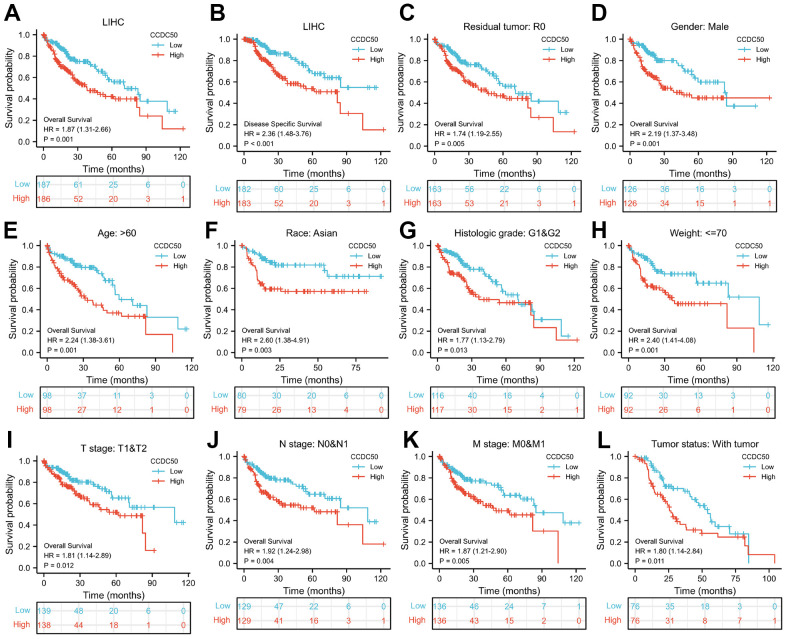
**Prognostic analysis of CCDC50 in HCC.** (**A**, **B**) The correlation between CCDC50 and OS, and DSS. (**C**–**L**) The correlation between CCDC50 and OS in different clinical subgroups of HCC, including residual tumour, gender, age, race, histologic grade, weight, TNM stage, AND tumour status.

As CCDC50 expression was up-regulated in HCC tissues, we explored the prognostic value of CCDC50 in HCC. The KM survival curve results showed that increased expression of CCDC50 related to poor OS and DSS in HCC (Figure 3A, 3B). We comprehensively analysed the relationship between CCDC50 expression and OS in HCC. High CCDC50 expression had worse OS in diverse subgroups of HCC, including residual tumour (Figure 3C), gender (Figure 3D), age (Figure 3E), race (Figure 3F), histologic grade (Figure 3G), weight (Figure 2H), TNM stage (Figure 3H–3K), and tumour status (Figure 3L).

**Figure 4:**
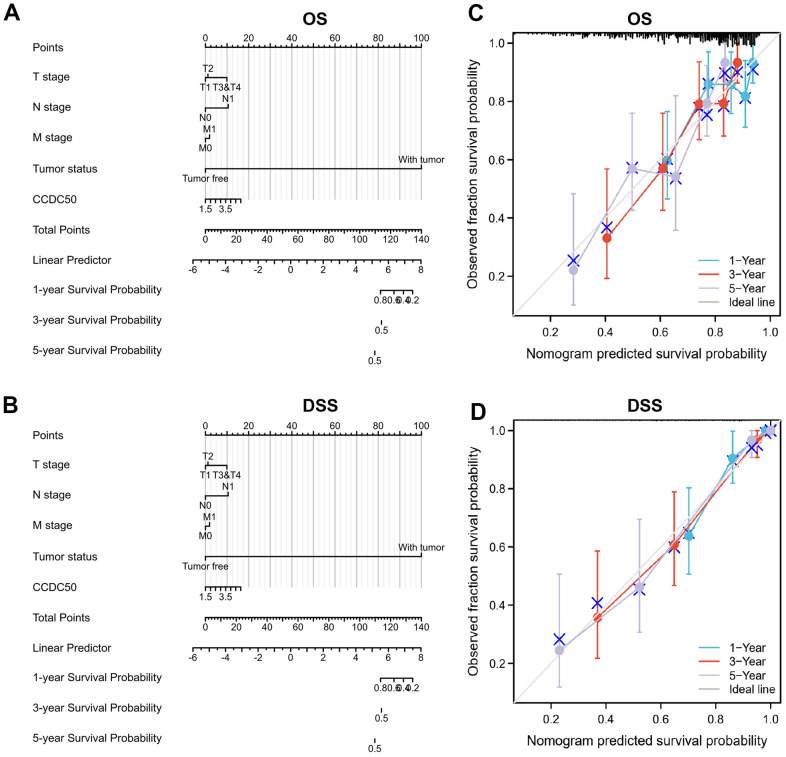
**Nomogram and calibration curve for predicting the probability of 1-, 3-, and 5-year OS, and DSS in HCC patients.** (**A**, **B**) A nomogram integrates CCDC50 and other prognostic factors in HCC from the TCGA data. (**C**, **D**) The calibration curve of the nomogram.

To examine whether CCDC50 can be used in HCC prognosis, we constructed a nomogram to predict the OS, and DSS in HCC patients and found that pathological stage and CCDC50 expression act as prognostic factors (Figure 4A, 4B and Tables 1–3). The calibration curves indicated that the nomogram can reliably predict the 1-, 3-, and 5-year OS, and DSS in HCC (Figure 4C, 4D). In summary, the model constructed above can optimize the prediction of different survival rates for liver cancer patients, and has high performance in prediction.

**Table 1:** Univariate and multivariate Cox regression analyses of different parameters of overall survival in liver hepatocellular carcinoma.

**Characteristics**	**Total (N)**	**Univariate analysis**			**Multivariate analysis**	
		**Hazard ratio (95% CI)**	**P-value**		**Hazard ratio (95% CI)**	**P-value**
T stage	370					
T1	183					
T2	94	1.428 (0.901–2.264)	0.129		1.594 (0.874–2.907)	0.128
T3 and T4	93	2.949 (1.982–4.386)	**<0.001**		1.694 (0.226–12.699)	0.608
N stage	258					
N0	254					
N1	4	2.029 (0.497–8.281)	0.324			
M stage	272					
M0	268					
M1	4	4.077 (1.281–12.973)	**0.017**		1.304 (0.310–5.480)	0.717
Pathologic stage	349					
Stage I and Stage II	259					
Stage III and Stage IV	90	2.504 (1.727–3.631)	**<0.001**		1.727 (0.237–12.611)	0.590
Tumour status	354					
Tumour free	202					
With tumour	152	2.317 (1.590–3.376)	**<0.001**		1.953 (1.226–3.110)	**0.005**
CCDC50	373	4.512 (0.270–75.472)	0.295			

**Table 2:** Univariate and multivariate Cox regression analyses of different parameters of disease-specific survival in liver hepatocellular carcinoma.

**Characteristics**	**Total (N)**	**Univariate analysis**			**Multivariate analysis**	
		**Hazard ratio (95% CI)**	**P-value**		**Hazard ratio (95% CI)**	**P-value**
T stage	362					
T1	180					
T2	92	1.619 (0.869–3.016)	0.129		1.459 (0.608–3.498)	0.397
T3 and T4	90	4.328 (2.583–7.251)	**<0.001**		14.188 (0.791–254.370)	0.072
N stage	253					
N0	249					
N1	4	3.612 (0.870–14.991)	0.077		9.081 (1.141–72.298)	**0.037**
M stage	268					
M0	265					
M1	3	5.166 (1.246–21.430)	**0.024**		1.997 (0.466–8.557)	0.352
Pathologic stage	341					
Stage I and Stage II	254					
Stage III and Stage IV	87	3.803 (2.342–6.176)	**<0.001**		0.332 (0.018–6.028)	0.456
Tumour status	354					
Tumour free	202					
With tumour	152	775790759.389 (0.000–Inf)	0.994			
CCDC50	365	0.073 (0.000–31.328)	0.397			

**Table 3:** Univariate and multivariate Cox regression analyses of different parameters of progression-free interval in liver hepatocellular carcinoma.

**Characteristics**	**Total (N)**	**Univariate analysis**			**Multivariate analysis**	
		**Hazard ratio (95% CI)**	**P-value**		**Hazard ratio (95% CI)**	**P-value**
T stage	370					
T1	183					
T2	94	2.017 (1.409–2.888)	**<0.001**		1.100 (0.694–1.742)	0.686
T3 and T4	93	2.798 (1.969–3.975)	**<0.001**		0.895 (0.212–3.785)	0.880
N stage	258					
N0	254					
N1	4	1.370 (0.338–5.552)	0.659			
M stage	272					
M0	268					
M1	4	3.476 (1.091–11.076)	**0.035**		1.462 (0.447–4.782)	0.530
Pathologic stage	349					
Stage I and Stage II	259					
Stage III and Stage IV	90	2.201 (1.591–3.046)	**<0.001**		2.043 (0.488–8.555)	0.328
Tumour status	354					
Tumour free	202					
With tumour	152	11.342 (7.567–17.000)	**<0.001**		15.140 (9.082–25.237)	<0.001
CCDC50	373	2.303 (0.186–28.565)	0.516			

### Gene mutation landscape of CCDC50 in HCC

**Figure 5:**
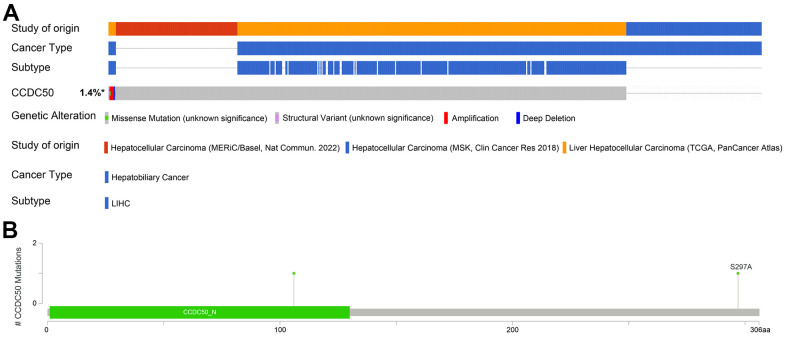
**Mutational analysis of CCDC50 in HCC.** (**A**) OncoPrint visual summary of the alterations in the CCDC50 gene. (**B**) The hot spots of mutations in CCDC50.

The mutational data of CCDC50 from the cBioPortal that showed genetic alterations in the CCDC50 gene were observed in only 1.4% of the HCC patients (Figure 5A), and 1 missense site was found between amino acids ‘0’ and ‘306’ (Figure 5B). These results indicate that genetic alterations of CCDC50 may not be the main factor affecting its prognostic ability.

### DNA methylation modulates CCDC50 expression in HCC

**Figure 6:**
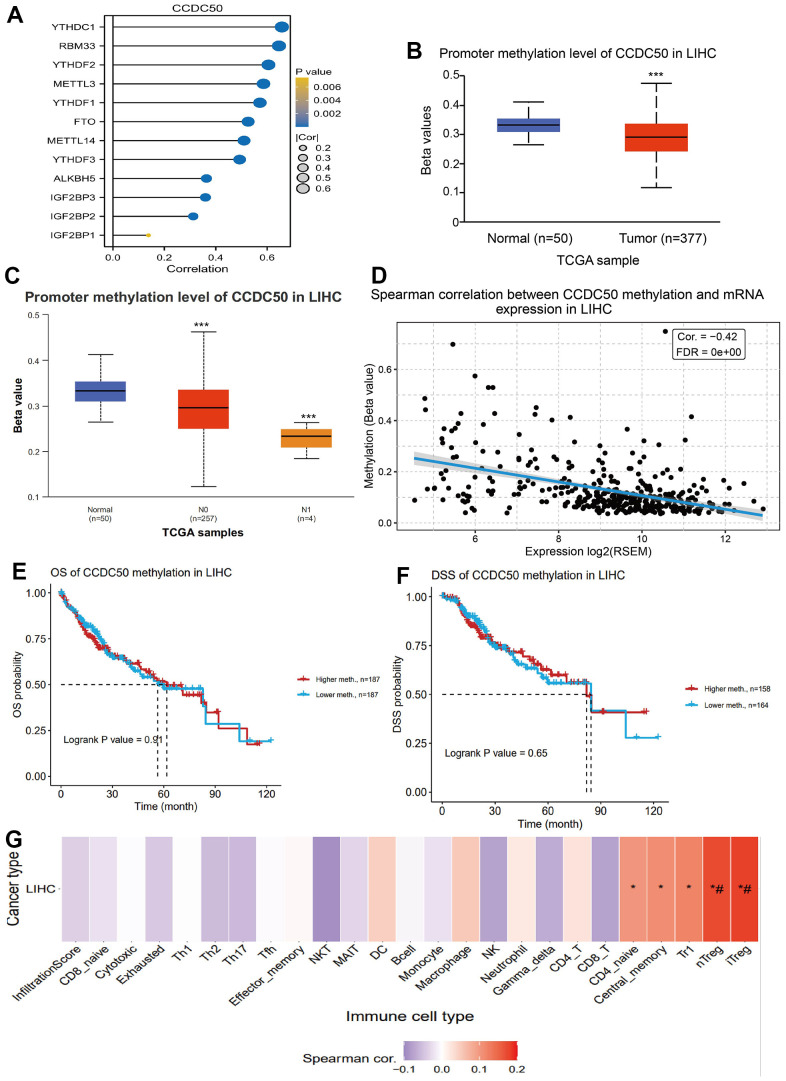
**DNA and RNA methylation analysis of CCDC50 in HCC.** (**A**) Correlation between m6A-related modulator genes and CCDC50 in LIHC. (**B**, **C**) The level of DNA methylation of CCDC50 in liver cancer tissue was significantly lower than that in normal liver tissue, and was negatively correlated with liver cancer metastasis. (**D**) The level of DNA methylation of CCDC50 was significantly negatively correlated with the expression of CCDC50 in HCC. (**E**, **F**) Correlation between DNA methylation of CCDC50 and prognosis in LIHC. (**G**) Correlation between DNA methylation of CCDC50 and the level of immune cell infiltration in LIHC. RSEM is a software for quantifying gene expression based on STAR sequence comparison. ns, p > 0.05; *p < 0.05; **p < 0.01; ***p < 0.001.

DNA methylation and m6A methylation play crucial roles in cancer progression. Therefore, it is crucial to reveal whether CCDC50 is regulated by DNA methylation and then abnormally expressed in hepatocellular carcinoma. First, we confirmed that CCDC50 was positively correlated with m6A regulatory factors expression in HCC (Figure 6A). Furthermore, we found that the level of DNA methylation of CCDC50 was downregulated in liver cancer tissue and was negatively correlated with liver cancer metastasis (Figure 6B, 6C). Finally, we found that the level of DNA methylation of CCDC50 was negatively related to the expression of CCDC50 in HCC (Figure 6D). However, the level of DNA methylation of CCDC50 did not affect the prognosis of HCC patients in TCGA LIHC datasets (Figure 6E, 6F). Interestingly, the level of DNA methylation of CCDC50 was positively correlated with the Tr1 and iTreg cell infiltration level (Figure 6G). These results partially suggest that DNA methylation may affect the infiltration levels of diverse immune cells by regulating the expression of CCDC50 in HCC.

### Biological functions of CCDC50 in LIHC

**Figure 7:**
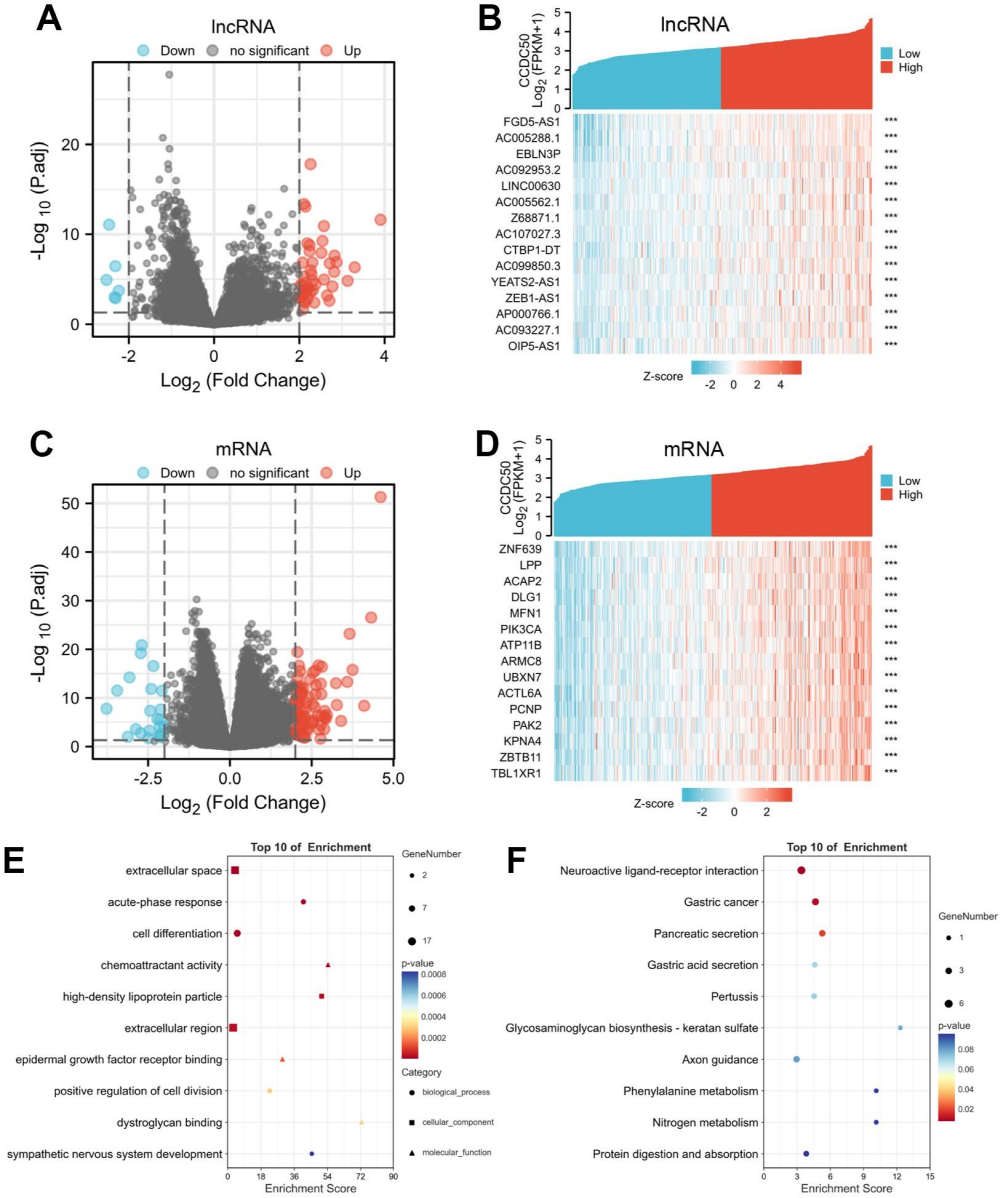
**Identifying differentially expressed genes (DEGs) between high and low expression of CCDC50 groups.** (**A**) The volcano plot of differential lncRNA profiles between CCDC50 high expression and CCDC50 low expression. (**B**) The heat map of the top 15 DEGs (lncRNA) between CCDC50 high expression and CCDC50 low expression. (**C**) The volcano plot of differential mRNA profiles between CCDC50 high expression and CCDC50 low expression. (**D**) The heat map of the top 15 DEGs (mRNA) between CCDC50 high expression and CCDC50 low expression. (**E**) The gene ontology term of CCDC50 analysis by using DEGs. (**F**) The KEGG term of CCDC50 analysis by using DEGs. ***p < 0.001.

Using the “clusterProfiler” R package, we performed functional annotations of CCDC50-associated differentially expressed genes (DEGs) in HCC patients, and 423 DEGs (mRNA and lncRNA) were captured, including 331 upregulated and 92 downregulated genes (Figure 7A–7D). The GO enrichment results showed that the DEGs were mainly involved in the extracellular space, acute-phase response, cell differentiation, chemoattractant activity, and epidermal growth factor receptor interactions (Figure 7E). KEGG results showed that the DEGs were mainly involved in the gastric cancer, pancreatic secretion, gastric acid secretion, pertussis, glycosaminoglycan biosynthesis-keratan sulphate, axon guidance, phenylalanine metabolism, nitrogen metabolism and protein digestion and absorption (Figure 7F).

**Figure 8:**
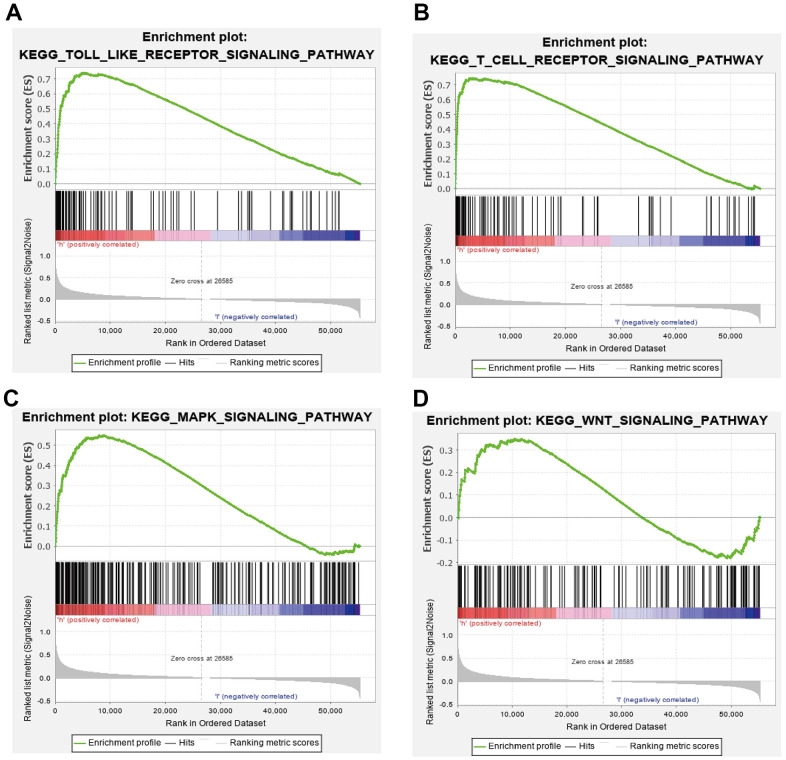
**GSEA of CCDC50 in HCC.** (**A**–**D**) The top GSEA results of CCDC50 in HCC.

GSEA results suggested that CCDC50 mainly participated in TOLL like receptor, T cell receptor, MAPK and wnt signaling pathway, chemokine signaling pathway (Figure 8A–8D). These results confirmed that CCDC50 regulated immune-related pathways and participate in the malignant progression of liver cancer.

### Immune cell infiltration of CCDC50 in HCC

**Figure 9:**
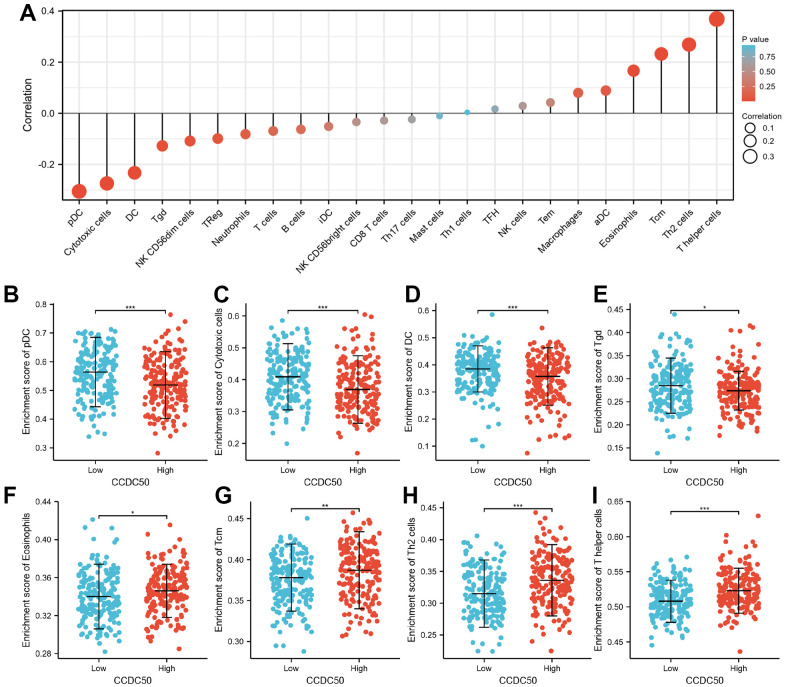
**The correlation between CCDC50 expression and the level of immune cell infiltration.** (**A**) The correlation between CCDC50 expression and the level of immune cell infiltration in HCC by using the TIMER database. (**B**–**I**) The correlations between CCDC50 expression and the level of pDC, Cytotoxic cells, DC, Tgd, Eosinophils, Tcm, Th2, and T helper cells. *p < 0.05, **p < 0.01, ***p < 0.001.

Immune cells have a critical role in cancer progression [25]. We used the TIMER database to reveal the relationship between CCDC50 expression and the infiltration levels of 24 immune cell types in HCC. The expression of CCDC50 significantly correlated with these 8 major immune cells in HCC (Figure 9A). CCDC50 expression levels showed a negative correlation with pDC (Figure 9B), Cytotoxic cells (Figure 9C), DC (Figure 9D), and Tgd (Figure 9E), and positive association with Eosinophils (Figure 9F), Tcm (Figure 9G), Th2 cells (Figure 7H), and T helper cells (Figure 9I). Due to the heterogeneity of tumor cells and immune cells, the expression of CCDC50 in hepatocellular carcinoma may be significantly different from the correlation between different immune cells. It is this difference that leads to tumor immune escape or the formation of tumor immune tolerance.

### Knockdown of CCDC50 suppresses the malignant phenotype of LIHC

**Figure 10:**
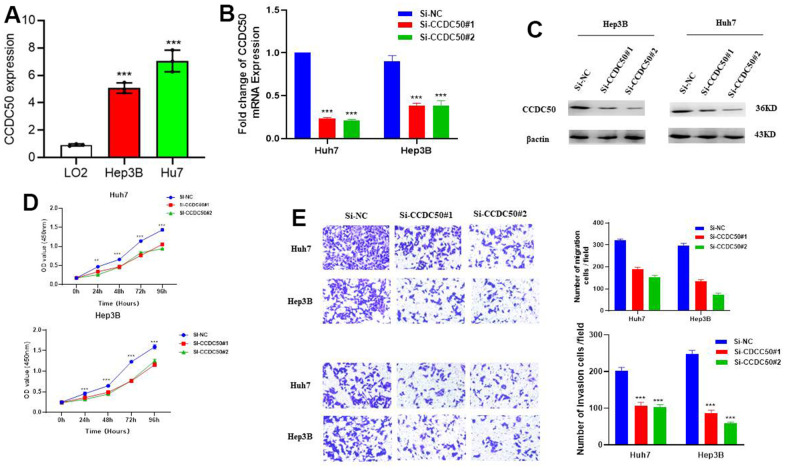
**CCDC50 promotes the proliferation, migration, and invasion of LIHC cells.** (**A**) The expression of CCDC50 in LIHC cell lines was examined via qRT-PCR assay. (**B**, **C**) The establishment of CCDC50 knockdown cell lines in Hep3B and Huh7 was verified via qRT-PCR assay and western blot. (**D**) The knockdown of CCDC50 dramatically inhibits the proliferation of Hep3B and Huh7 cells, examined via cell counting kit-8 assay. (**E**) The knockdown of CCDC50 dramatically inhibits the migration and invasion abilities of Hep3B and Huh7 cells. Data are presented as the mean ± SD of three independent experiments. **p < 0.01, ***p < 0.001.

*In vitro* cell biology functional experiment validation of the functional role of CCDC50 in HCC was performed. Compared to LO2, a human normal hepatocyte, CCDC50 was highly expressed in LIHC cell lines (Figure 10A) and inhibited by siRNA in Hep3B and Huh7 cells. QRT-PCR and western blot were employed to examined the knockdown efficacy (Figure 10B, 10C). As expected, CCDC50 knockdown inhibited the proliferation and migration abilities of Hep3B and Huh7 cells (Figure 10D, 10E).

### Cancer drug sensitivity analysis of CCDC50

**Figure 11:**
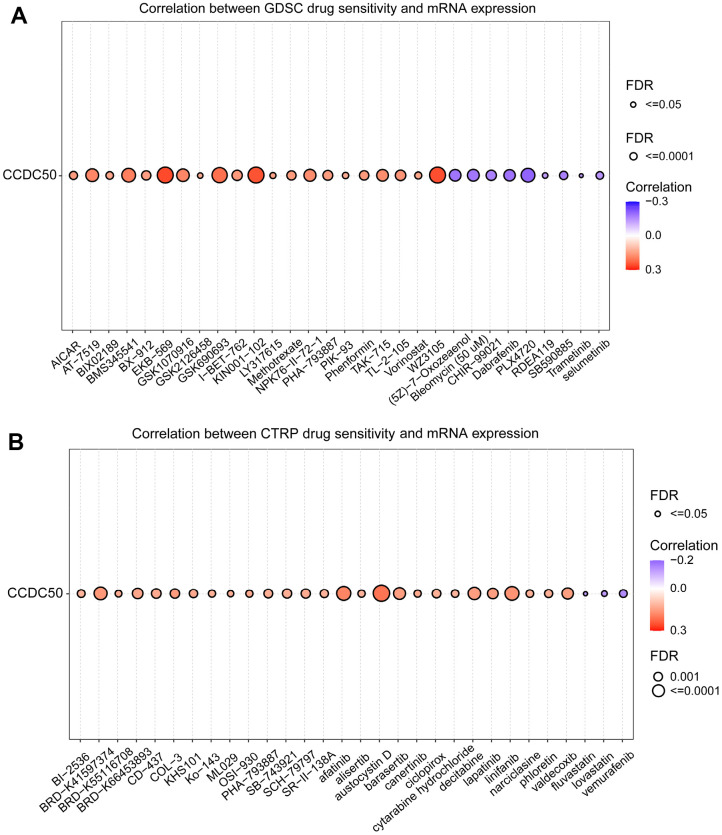
**Analysis of the correlation between CCDC50 expression and drug sensitivity in various cancers.** (**A**) The correlation between CCDC50 expression and drug sensitivity in various cancers was analysed via the GDSC database. (**B**) The correlation between CCDC50 expression and drug sensitivity in various cancers was analysed via the CTRP database.

In the GDSC database, the expression of CCDC50 positively related to the following drugs: EKB-569, WZ3105, KIN001-102, AT-7519, GSK690693, BMS345541, AT-7519, GSK1070916, NPK76-II-72-1 and TAK-715. However, it was negatively correlated with the following drugs: PLX4720, dabrafenib, (5Z)-7-Oxozeaenol, bleomycin, SB590885, CHIR-99021, selumetinib, TGX221, PD-0325901, AG-014699 and piperlongumine (r < −0.13, p < 0.0001) (Figure 11A). In the CTRP database, the expression of CCDC50 positively correlated with the following drugs: SR8278, GSK-J4, austocystin D, afatinib, linifanib and BRD-K41597374 (r > 0.16, p < 0.0001). However, it was negatively associated with vemurafenib, MLN2480, fluvastatin and lovastatin (r < −0.13, p < 0.0001) (Figure 11B). Due to the heterogeneity of tumor cells, there are certain differences in the correlation between CCDC50 and different drugs in different tumors, which may have a certain impact on different tumor treatments. These results show that CCDC50 is significantly related to drug sensitivity in diverse cancer cell lines, and it has the potential to be a promising cancer therapeutic target.

## DISCUSSION

Prevention and treatment are important scientific problems for HCC patients. The bioinformatic analysis is critical to compare the heterogeneity among different tumours and identify novel cancer biomarkers and therapeutic targets [26, 27]. In the early stage, we also reported the relationship between CDH11 and malignant progression of gastric cancer through the public cancer database [28]. CCDC50, as a negative regulator of IFN signalling, is ubiquitously expressed in human tissues [9]. It has been reported CCDC50 is involved in the progression of renal clear cell carcinoma and mantle cell lymphoma [10, 29]. Further, CCDC50 could regulate Ras signalling pathway and promote mice hepatocellular carcinoma [12]. However, until now, no studies have assessed whether CCDC50 is associated with human HCC prognosis.

In this study, we firstly reported that CCDC50 expression was up-regulated in HCC. In addition, our results indicated that high CCDC50 expression correlated with poor prognosis in HCC, the calibration curves indicated that a nomogram is reliable to predict the 1-, 3-, and 5-year OS, and DSS in HCC. To better understand the role of CCDC50 in HCC, the KEGG enrichment analysis was performed, and it indicated that the DEGs in HCC were primarily involved in the neuroactive ligand-receptor interaction, gastric cancer, pancreatic secretion, gastric acid secretion, pertussis, glycosaminoglycan biosynthesis-keratan sulphate, axon guidance, phenylalanine metabolism, nitrogen metabolism and protein digestion and absorption.

A previous study found that 25% of HCC samples expressed inflammatory biomarkers [30]. Tumor immune cell infiltration might be associated with CDC50 expression, as shown in our study. We showed that CCDC50 correlated with these 8 major immune cells in HCC. CCDC50 expression levels showed a negative correlation with pDC, Cytotoxic cells, DC, and Tgd, and positive association with Eosinophils, Tcm, Th2, and T helper cells.

Based on the comprehensive bioinformatics analysis of liver cancer related datasets and the results of our cell biology functional experiments, we can preliminarily conclude that CCDC50 may play a role as an oncogene in the progression of liver cancer, promoting the malignant progression of liver cancer cells. In the future, it can be used as an important liver cancer biomarker and a potential therapeutic target.
